# Adolescent Perception of Mental Health: It’s Not Only about Oneself, It’s about Others Too

**DOI:** 10.3390/children10071109

**Published:** 2023-06-25

**Authors:** Justė Lukoševičiūtė-Barauskienė, Monika Žemaitaitytė, Vaida Šūmakarienė, Kastytis Šmigelskas

**Affiliations:** 1 Department of Health Psychology, Faculty of Public Health, Medical Academy, Lithuanian University of Health Sciences, LT-47181 Kaunas, Lithuania; 2 Faculty of Public Health, Health, Research Institute, Medical Academy, Lithuanian University of Health Sciences, LT-47181 Kaunas, Lithuania

**Keywords:** adolescent, mental health, qualitative research, relationships, emotions, behavior, risk factors

## Abstract

Adolescents are at an increased risk of mental health problems due to the natural processes of development and maturation. Given that their mental health is mainly assessed by adults and not by the adolescents themselves, the purpose of this study is to reveal adolescents’ perceptions of mental health. Semi-structured, in-depth interviews were conducted, and an inductive qualitative approach with thematic analysis was used. The qualitative study consisted of 19 adolescents aged 11–17 years. Five themes were identified: (1) What does mental health mean to you? (subthemes: realm of emotions, customary behavior, and relationships with others); (2) needs (subthemes: communication and support, self-expression and freedom of decision-making, and a safe and personal environment); (3) risk factors (subthemes: un-healthy relationships and social media dangers); (4) red flags (subthemes: self-exclusion from social life and self-destructive behavior); and (5) role of mental health professionals (subthemes: attentiveness to and proper pace for adolescent and acceptance of adolescent’s life in its entirety). This study revealed that adolescents view their mental health not only from their own emotional and behavioral perspectives but also through the prism of relationships with other people.

## 1. Introduction

The World Health Organization defines mental health as “a state of well-being in which the individual realizes his or her abilities, can cope with the normal stresses of life, can work productively and fruitfully, and is able to make a contribution to his or her community” [1]. It is not un-common for this concept to be expanded to refer to mental health as a continuous construct, where mental health and illness are at opposite ends of a single continuum. Most commonly, the term “mental health” defines a person’s overall psychological state of well-being, including the distinction between mental health and mental illness. By this definition, individuals with good mental health have high levels of vigor and low distress [2]. In recent years, the context of the global COVID-19 pandemic has led to increased attention to mental health and well-being, in part due to increasing mental health problems among children and adolescents [3]. Therefore, mental health has become one of the major priorities for adolescence-related research, policy, and health in general.

First, adolescence is associated with many changes, starting with a cascade of developmental changes in the young person’s body, such as rapid physical growth, metabolic development, sleep, and circadian changes, as well as puberty [4]. This transition from childhood to adulthood involves behavioral changes, such as sensation seeking, re-orientation of attention and motivation, and new experiences—changing social contexts, roles, and responsibilities [5]. Due to these changes, adolescence is a developmental period of vulnerability and opportunity, so mental health during this sensitive period is critical to overall health and well-being. On the one hand, adolescents are at higher risk of psychological distress than younger children and adults [6], because the risk of death and disability increases due to untoward events such as accidents, depression, substance use, unwanted pregnancies, and other health-related behaviors that contribute to mental health outcomes in later life [5]. As a result, adolescence is often identified as a stage of increased vulnerability to mental health problems—as many as 3/4 of adults who have ever suffered from a mental health condition report experiencing the first symptoms during adolescence [7]. Regardless of such vulnerabilities due to changes, adolescence can also be a period full of opportunities for psychosocial growth [6].

Thus, while growing up, adolescents also acquire more self-expression and psychosocial abilities [8], develop more mature relationships, express their authentic identity, take more responsibility for their behavior, and search for and solve questions related to their direction in life [6]. Nevertheless, problems such as depression, anxiety, attention deficit hyperactivity disorder (ADHD), and pro-social behavior are central constructs in research on adolescent psychosocial development [9,10]. Common measures of mental health such as PHQ-9, GAD-7, RCADS-25, and WHODAS 2.0 also evaluate depression, anxiety, or impairment [11]. Not surprisingly, in the context of the pandemic, the attention of researchers has remained focused on the most common symptoms—anxiety, depression, sleep, and appetite disturbances—but has also affected social interactions [12]. The systematic review by Panchal et al. [13] revealed that anxiety and depression symptoms, irritability, and anger were the most common symptoms during the lockdown in children and adolescents. Moreover, studies show that a good parent-child relationship can protect against anxiety and depression [13].

Although international health policies are increasingly focusing on the relevance of the young person’s voice and empowering the young person to manage their care and treatment, clinicians in child and adolescent mental health often rely on reports from parents, teachers, or other professionals about a young person’s needs and treatment progress [12,14]. Therefore, it is important to hear adolescent opinions about issues related to their mental health, which can provide insights for interventions and policies aimed at improving their mental health [15]. Since children and youth are competent reporters of their health needs, the use of self-report measures is an effective way to engage young people in mental health treatment [14].

Given that adolescents’ mental health is mainly assessed by adults and not by the adolescents themselves, a more immediate, direct understanding of adolescents’ direct thoughts could provide additional comprehension about the overall understanding of their mental condition and how to improve it. A better understanding would provide more opportunities to empower adolescents by helping them take certain actions as well as seek help. To get a broader understanding of the adolescent perspective, this study used a qualitative design and aimed to reveal adolescents’ perceptions of mental health as a concept.

## 2. Materials and Methods

This study was approached using a qualitative research strategy. The target group was 11–17-year-old schoolchildren of both genders. The participants were collected using convenience sampling with an emphasis on diversity in terms of age, gender, and urban/rural residence.

**Figure 1:**
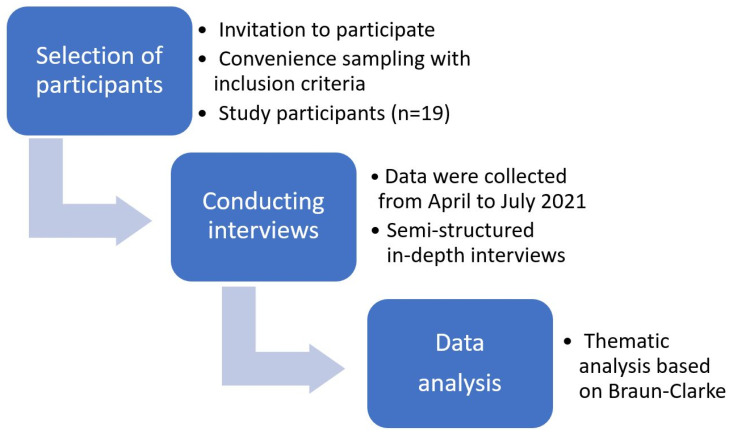
The research process of the qualitative study.

The design of the study was constructed by this article’s authors (Figure 1). The permission to conduct this study was obtained from the Ethics Committee for Biomedical Research (No. BE-2-41; 4 April 2021). The interviews and their content were anonymous, written informed consent of participants and their parents was required. Invitations to participate in the study were disseminated on social media sites, weblogs, and organizations of adolescents and their parents. The inclusion criteria were:

- age 11–17 years old (grades 5–11);
- ability to speak, write, and understand the Lithuanian language;
- written consent of the adolescent to participate in the study;
- written consent of both parents for an adolescent to participate in the study.

The participants were encouraged to talk by asking them open-ended questions in one-on-one interviews. All researchers were practicing psychologists and/or worked at universities as researchers in the field of child and adolescent psychology. The total sample consisted of 19 adolescents: 10 girls and 9 boys from various urban and rural locations (Table 1).

Data were collected from April to July 2021 through in-depth interviews. Due to COVID-19 restrictions, the participants could opt for in-person or online interviews (11 and 8, respectively). The semi-structured interviews were audio-taped and transcribed verbatim. The median duration of interviews was 22 min. 

The interview had one main question: “Each person understands mental health differently, individually. I would like to hear from you what you think about mental health. Please, describe what mental health means to you?”. If necessary, additional questions were also asked: “How would you describe your mental health?”, “What people help you maintain good mental health?”, “What do you do yourself to maintain good mental health?”, and “What harms your mental health?”.

The interviews were audio-taped and transcribed verbatim using the oTranscribe web app (https://otranscribe.com/, accessed on 3 May 2023).

A thematic analysis method was applied based on Braun and Clarke’s analysis [16]. The initial codes, categories, and themes were reviewed by all co-authors to validate the themes and subthemes.

## 3. Results

**Figure 2:**
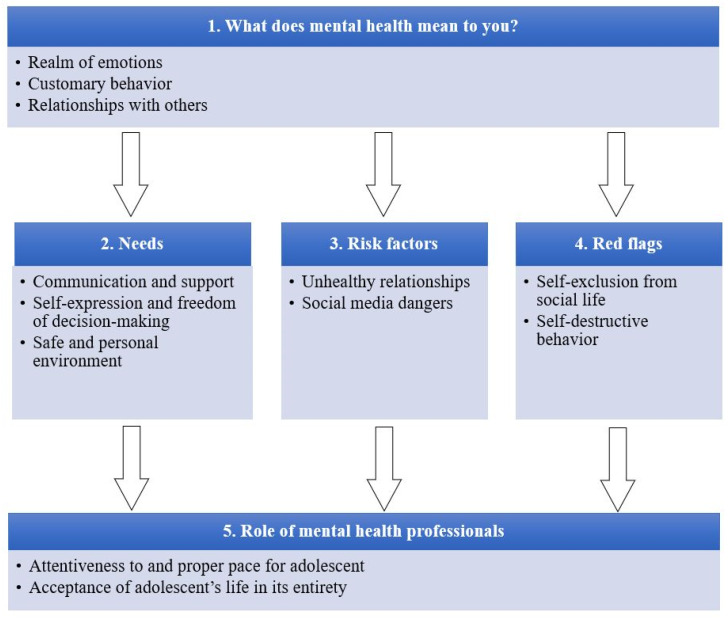
Thematic map: mental health perceptions among adolescents.

Based on the interviews with participants, five themes were identified (Figure 2). Every theme is described in detail below, along with subthemes. The most eloquent quotations are also presented with brackets indicating the participant’s gender, age, number of interviews, and segment.

### 3.1. What Does Mental Health Mean to You?

During interviews, the study participants tried to reveal how they perceive the concept of mental health. They interpreted the construct from an emotional perspective, foremost, but also through behavioral aspects and their relationships with others.

Realm of emotions (16/19). During the conversations, most frequently, the adolescents started sharing their perceptions relating mental health to emotions. They shared both positive and negative emotions that they could experience internally or express externally. The following positive emotions were most frequently mentioned: happiness, joyfulness, a positive attitude, a good mood, empathy, and not feeling angry or miserable. “<…> a person is happy and, umm, well, they are happy, satisfied with everything” (girl, 15 years, I9-S2). The negative emotions reflected during the interviews were the following: sadness, anger, depression, anxiety, nervousness, bad mood, and pessimism. Additionally, some adolescents defined mental health as the system in charge of emotions, “I understand mental health as a system that is responsible for a person’s emotions, and if it malfunctions, emotions also malfunction” (boy, 17 years, I4-S1). Moreover, some participants suggested that good mental health implies a proper expression and control of emotions: “<…> one shows adequate emotions, and they are not distorted in any way or they are not wrong emotions in certain situations” (girl, 14 years, I2-S4) or “They sometimes say you have mental health disorders, or that you are not able to control your emotions” (boy, 17 years, I4-S2).

Customary behavior (16/19). The majority of study participants associated mental health with behavioral expression. They emphasized that an adolescent with good mental health should not stand out from the people around them; they should respond to situations similar to their peers and should conform to a certain norm established in society: “You react to most things with the same emotions as most people. And, umm, that is what is called ‘a norm’ <…> if, say, my mental health is okay, then, similarly, he should, well, he should react in the way that I do” (boy, 17 years, I4-S2). When speaking about customary behavior, the adolescents provided multiple examples and situations, primarily reflecting on their learning environment, academic performance, and ability to learn at a similar level with their classmates: “<…> if one is learning something, one is learning at the same pace as all the others, as most of the others. And if one is, so to say, not mentally okay, then it may be a bit more difficult for them and a person may somewhat get left behind” (girl, 12 years, I11-S2). In addition, such standards of behavior include polite behavior in public (for example, no fighting, no insulting, no bullying, no hysteria, and no laughing at serious things), conscientiousness, and comprehension based on their age. Finally, some referred to being just not eccentric: “<…> not being weird, maybe even. Just ordinary” (girl, 11 years, I3-S2).

Relationships with others (16/19). From an adolescent perspective, relationships are an integral part of mental health—more than half of adolescents participating in interviews spoke about this aspect. When pondering on relationships as a feature of mental health, they specified relationships with their parents, friends, and teachers. They perceived mental health as communication with others, the proper way of speaking (voice, volume, and body language), the ability to accept the opinion of others, to listen to the interlocutor, to help others, or ask for help. Some adolescents also mentioned that having conflicts and enemies is an indicator of poor mental health: “<…> gets involved in multiple conflicts, has conflicts… with other children <…>” (girl, 11 years, I3-S5).

### 3.2. Needs

This study has also identified the most important needs of adolescents that are often ignored by the surrounding population but are relevant for adolescent mental health. Here, the participants expressed the need for communication and support, self-expression, and a safe and personal environment—the needs that are crucial during adolescence.

Communication and support (18/19). This subtheme came up in nearly all interviews. The study participants highlighted the need to communicate, to be heard, to be appreciated, and to get attention and support: “<…> we are created to communicate with other people” (girl 14, I14-S3), and “<…> a person cannot be lonely” (girl, 17 years, I1-S10). A good relationship with one’s parents was among the most frequently mentioned factors: “The main thing, I don’t know, probably, just like for everyone else, is family. Actually, I am really happy that I am in a very good relationship with my parents. If something starts annoying me, I don’t know, or if things seem to be getting out of control, or simply something is not right with my life, I can always approach them, talk to them” (girl, 17 years, I1-S10). To some extent, the relationship may not indicate direct communication, but rather a perceived sense of support: “Sometimes, I do not even have to say anything to them” (girl, 17 years, I1-S10). Adolescents also mentioned the relationships with peers and classmates with whom they could talk, and whose ideas and problems were similar. From the perspective of adolescents, belonging to a peer group and frequent meetings with friends helps improve their mental health and constitutes one of their key needs. Other significant people mentioned were teachers, grandparents, cousins, other relatives, and sometimes even strangers, and all people who are together with adolescents, who listen to them, are supportive, provide considerable attention, or just pay a compliment. Another resource for better mental health in adolescents is pets: the significance of this relationship was highlighted through the need to take care of them and to spend time together, which has an overall positive and calming effect.

Self-expression and freedom of decision-making (13/19). This need was reflected in over half of the conversations with study participants. The adolescents shared that having dreams and making them come true is highly important for good mental health: “Those goals of mine, they somehow motivate me, and then I feel good” (girl, 17 years, I1-S16). Being engaged in one’s favorite activities and having new experiences also have a positive effect on mental health. The participants spoke about various activities that were helpful in improving their mental health. Usually, these are creative activities such as sewing, playing a musical instrument, or drawing, through which they are also able to express their emotions. Furthermore, the adolescents mentioned more intellectual activities, e.g., reading, learning, or problem-solving based on books or movies. Other participants emphasized the need to behave and make decisions freely and independently without others telling them how: “When that help is forced onto you, and you have not even asked for it, you do not actually need it, then, well… well, then it is not really needed. <…> When you are offered a detailed plan that will make things alright if you follow it. Thank you… thanks… No! I do not know. I am an independent person. I do not need others to tell me what to do and where to do, and how, and with whom, and why” (girl, 17 years, I19-S10).

Safe and personal environment (11/19). Slightly more than half of the interviewees revealed the need for safety and peace of mind in their current environment. The adolescents mentioned that for good mental health, it is crucial to feel safe with the people one lives with. A safe school environment was also indicated because adolescents tend to spend most of their day at school. One participant said that her mental health was poor because: “I feel good neither at school nor at home” (girl, 17 years, I1-S16). Fear of being scolded was also indicated: “<…> somehow you think that what you are doing is bad, even though you are doing everything correctly. You think that you will be told off although you have done nothing bad” (boy, 12 years, I7-S4). The need for peacefulness and personal, private space was also emphasized: “<…> those most important people can simply be too annoying with their wish to know how they could help you, even though you do not need their help and just want to stay alone for a couple of days, left in peace. How can I put it–to retreat for a while, think everything over, and then get back on the track” (boy, 15 years, I10-S10).

### 3.3. Risk Factors

The data collected during the study has enabled us to define certain risk factors for adolescent mental health. Based on the ideas expressed by participants, harmful relationships and social media are most damaging to their mental health.

Unhealthy relationships (14/19). The study participants mentioned harmful relationships as one of the risk factors for adolescent mental health. According to adolescents, this involves having bad friends and enemies, teasing, calling names, or bullying from peers. They also mentioned that teasing can be both face-to-face and online. Moreover, the study revealed that bullying from teachers, impolite comments, and underestimation also have a damaging effect on adolescents’ mental health. When it comes to the family environment, there must be no bad feelings between parents and siblings because conflicts with the closest people also harm adolescents’ mental health.

Social media dangers (6/19). Social media was identified by adolescents as another factor with a negative effect on mental health. Long screen time (mobile phone, computer, TV, etc.) and the information presented there are potentially harmful since adolescents cannot assess them critically or select the most reliable sources of information: “Say, if you take YouTube. If you are following some content developers there, and they just do some negative stuff, this may damage your understanding of the world” (boy, 17 years, I4-S11). Furthermore, the effect of social media on the self-esteem of an adolescent was also highlighted: “<…> adolescents like identifying themselves with others so that… when you are watching photos or things like that on social networks, and… you see that someone is doing well, or that they are just succeeding in something, or that they are okay, while you have not probably achieved such things. This just goes to your head, even though this, ummm, should not really mean much to you” (boy, 17 years, I4-S11). One girl also stressed that social media is often more dangerous to girls because this might affect how they see their own bodies or perceive what is beautiful: “<…> a lot of girls have this bad attitude towards their bodies, as they say. That she has to be, that she has to look in a certain way, that she has to be pretty. That she has to be suitable for others, for that whole world, the environment” (girl, 14 years, I14-S11).

### 3.4. Red Flags

During the conversations with adolescents, certain aspects were observed that should draw the attention of others and that signal poor mental health. Help and attention from the surrounding people are required when an adolescent is in social exclusion, introverted, or behaves destructively.

Self-exclusion from social life (12/19). More than half of the study participants said that separation from others is a highly important factor that has a damaging effect on adolescents’ mental health. When speaking about self-exclusion, the adolescents provided the following examples: sitting on a bench without communicating with anyone, sitting somewhere in a corner all alone, spending time in their room without communicating even with family members, or not going out with friends. According to participants, loneliness, avoidance of any communication, ignoring others, or trying to stay invisible constitute the risk factors for mental health: “<…> sitting there in the corner, not showing oneself to anyone–just trying to stay invisible so that no one would notice him. So… hidden” (girl, 11 years, I3-S6); “<…> he just started ignoring and not speaking with others; I guess it could spot that he really needs help and assistance through that difficult period” (boy, 15 years, I10-S6). The participants believe that such behavior indicates poor mental health and has a negative impact. Moreover, doing nothing and not learning during lessons were also mentioned by adolescents as the type of behavior that has a damaging effect on mental health: “<…> he did not even try to do anything in lessons, and for teachers… they blamed teachers for their wrongdoings and sought recognition among friends” (boy, 17 years, I12-S6).

Self-destructive behavior (12/19). By this, the adolescents had in mind various types of risky behavior. Addictions—such as smoking, drinking alcohol, or drug use—were among the most frequently mentioned examples. In addition, adolescents noted destructive behavior directed towards others, such as fighting with peers, reckless, impulsive behavior, or behavior that can hardly be understood or expected by others. Some adolescents (3 out of 19) mentioned suicidal thoughts, thoughts about self-harm, and attempts to commit suicide (by cutting wrists or using drugs): “If you found him there with his wrists cut open, or with a heroin needle” (girl, 17 years, I19-S6).

### 3.5. Role of Mental Health Professionals

The last theme revealed the role of mental health specialists working specifically with adolescents, where personal attention to an adolescent, a proper pace, and a holistic approach when assessing their mental health are worth considering.

Attentiveness to and proper pace for adolescents (14/19). The study participants mentioned that a specialist should not hurry, but rather start a conversation with simple, more optimistic questions (e.g., about their day) or pleasant things (e.g., about pets). Only then should they move on to more complicated questions regarding emotions or problems. Adolescents argued such a sequence of conversation by their difficulties speaking about themselves openly, problems in the identification of their own emotions, or even concealment of mental state: “I do not really think that when mental health is poor or bad it is possible to notice it every time <…> if a person wants to hide it, they will hide it, and you will never suspect that” (girl 17 years, I19-S5); or “<…> there are people who suffer from poor mental health, but they somehow try to cover this up” (girl 17 years, I1-S4). Nonetheless, adolescents trust mental health professionals and think that they can offer them the best possible help. The study participants also said that for having a more casual conversation, specialists should try to identify themselves with an adolescent: “[specialists could] remember their own adolescence troubles, their own adolescent days” (girl, 16 years, I17-S7). It is very important to ask adolescents how they are feeling instead of following the predominant stereotypes in society—especially when speaking about the mental health of young boys.

Acceptance of the adolescent’s life in its entirety (14/19). When asked about the key areas reflecting adolescent mental health, the adolescents indicated a large variety of them. In their opinion, a holistic approach is important, i.e., their childhood experiences have to be analyzed in great detail, as well as their home environment and living conditions, various aspects of their upbringing, and their relationship with parents and pets. The participants also shared much about the relevance of friends—having them, keeping good relationships, social support, and even the mental condition of friends. In addition, the school environment was indicated because adolescents’ behavior at school reveals much about their mental health. Regarding behavioral patterns, it is crucial that adolescents have favorite activities, their own leisure time, and no addictions. According to study participants, all these factors are important when assessing adolescents’ mental health. These aspects should not be considered in isolation; therefore, specialists should pay attention to them during a conversation with adolescents.

## 4. Discussion

One of the public health issues is mental health problems suffered by young people, so it is crucial to hear the views of adolescents about the things that affect their lives [17]. Since there is a lack of research on the perception of mental health among adolescents, we aimed to qualitatively assess the attitudes of adolescents on this topic. The thematic analysis outlined that adolescents’ mental health is related to acceptable, appropriate emotions and their expression, undifferentiated behavior with others, and is also closely linked to the quality of social relations. These findings partly support the view of mental health care professionals and scientists that mental health is not only the absence of symptoms but also a comprehensive quality of life and functioning [18].

However, in our study, a slightly different perception of the mental health of the adolescents themselves emerged: in addition to the aforementioned emotional and physical health, the participants of this study emphasized relationships with others and one’s behavior. Specifically, adolescents defined their understanding of mental health in terms of non-differentiating behavior that conforms to societal and peer norms. On the one hand, the expressed approach is confirmed by the creation and adaptation of classifications of mental disorders (DSM, etc.), which define norm-compliant, adaptive behavior, and deviation from being considered a mental disorder. On the other hand, adolescents at this stage of their lives often want to stand out from other peers through communication, self-expression choices, different, un-usual reactions to social situations, experimentation, or different levels of risk [19].

The importance of relationships was an un-expected aspect of perceptions of mental health among adolescents. In this study, it was one of the main pillars of mental health since it was mentioned and approached from various perspectives in the interviews, even though theoretically, relationships are not a direct constituent of mental health. Our study participants specified relationships with their parents, friends, teachers, or even strangers. The importance of peer communication and social relationships in adolescence is demonstrated in previous studies, which indicate adolescents’ transition towards independence, spending less time with family but more with friends and romantic partners [20]. Social support from friends was found to be associated with better mental well-being, and fewer anxiety and depression symptoms [21].

Our study participants talked about independence from parents and their instructions. These results are in line with other researchers’ findings that one of the most challenging tasks in adolescence is learning to find a balance between independence and dependence within social relationships [22]. However, recent studies support the persisting relevance of family support in adolescence—they found that higher family support relates to less bullying and fighting [23], and fewer depression and delinquency outcomes [24].

Even though adolescents often seek to be independent and to separate from their parents (according to their development and the tasks related to it), when facing difficulties, attention, connection, and the opportunity to talk with parents are important. This is supported by a systematic review by Radez et al. [25], where it was found that young people prefer to discuss their mental health difficulties with family members and friends rather than with professionals. Communication with parents and their support could be indirectly associated with adolescents’ mental health and could be a protective factor (for example, in cases of bullying, depression, anxiety symptoms, conflicts at school, etc.).

Overall, the role of adults in adolescents’ mental health also extends to teachers. Studies on bullying experiences confirmed that social support from family and teachers reduces the likelihood of depressive and anxiety symptoms, while higher social support from the family increases the probability of better subjective well-being [26].

Slightly less than relationships with adults, those with agemates were also mentioned by adolescents. Here they emphasized that harmful relationships are one of the risk factors for adolescent mental health, especially bullying and having bad friends (or even enemies). Studies show that bullying victimization during childhood affects not only short-term but also long-term well-being [27,28]. Bullying, cyberbullying, and fighting at school are very common concerns about peer violence among adolescents. The existence of bullying at school harms the school experience and violates the rights of children to learn in a safe environment. Another study confirmed that both bullying at school and cyberbullying or victimization were associated with increased depression among adolescents [29].

Adolescents also expressed their concerns about the impact of social media on mental health. In previous studies of adolescent samples, social media use was commonly associated with decreased face-to-face human interaction, addictive-like behaviors, social pressure experienced by adolescents due to social comparisons, and even suicidal behavior [30]. Our study participants envisaged such negative aspects of social media as the harm of long screen time, misleading information (especially mentioned by boys), and potentially harmful effects on self-esteem and self-image (especially for girls). These findings support those by Valkenburg et al. [31] in their literature review. Most analyzed studies show that frequent social media use in adolescence could be associated with poorer mental health [32,33], depressive symptoms [34], anxiety, or distress [35]. Additionally, some studies do not find any significant associations between social media and adolescent mental health, or the results are inconsistent [36,37].

Another facet of poor adolescent mental health reported by our study participants was self-destructive behavior, implying smoking, drinking alcohol, or drug use. Even though it is a direct behavioral component, previous research shows that it relates to mental health. For instance, Ferreira et al. [38] found that smoking and alcohol consumption are associated with psychological problems among adolescents. Other studies also confirm that alcohol use among youth is related to several specific emotional (such as anxiety, depressiveness, and attention problems) or behavioral syndromes (such as aggressive behavior, rule-breaking problems, and social problems) [39].

In our study, some adolescents mentioned suicidal thoughts, thoughts about self-harm, and attempts to commit suicide. Such self-harm behavior shows a danger not only to mental but also to physical health. Studies confirm that having thoughts about suicide or attempting suicide significantly correlates with the number of role models seen as smoking or drinking [40]. It can be assumed that self-destructive behavior is possibly one of the coping mechanisms of adolescents, especially if they do not feel like living in a safe environment. There is empirical evidence that peer influence is important in the development of antisocial behavior during adolescence [41].

Young people who do not feel well in their home environment may seek support and strive to belong to a peer group (often also distinguished by behavior). A recent study revealed that disengagement coping predicted suicidal ideation, past attempts, and depressive symptoms among youth [42]. Moreover, young people (16–19 years old) who do not have a safe home environment tend to have more risky behaviors. Homeless young people were more likely to report substance use, legal, academic, and mental health problems [42].

Thus, our study findings that adolescents associate risk behaviors, bullying, suicidal intentions, and other negative factors with their mental health indirectly demonstrate the relevance of safety in adolescent life.

The associations with bullying indicate the relevance of a safe environment and communication to the mental health of adolescents. In our study, the participants shared their need to have a safe place at school and at home. Their idea was that the environment should be free of bullying and fighting and have private space if one needs it. Researchers show that collaborative, proactive anti-bullying policies could foster a safe environment for youth [43]. For adolescents, how they feel fitting at school in terms of people and studies is an important factor in their well-being. A qualitative study in Australia showed that school could be not only a physical place to be, but also a caring and safe place; they reported that school provides identity, is a place for personal and social relationships, is a place to contribute, is a place to learn, and is a place to be recognized [44].

A final theme that emerged from our research was the role of mental health professionals. Previous research shows that young people seeking help may have fears of being taken away from their parents [45], of losing status among peers [46], or of making their family angry or upset [47]. In our study, the adolescents also expressed concerns regarding how they could be approached by mental health professionals. They shared the relevance of a specific manner they would expect from professionals providing mental health care, mainly attentiveness, acceptance, and equal relationships. A systematic review of studies about children’s and adolescents’ access to professional help for mental health problems also adds that young people seek help more often if they feel respected, listened to, and if the problems and emotions they express are taken seriously and not judged [25].

When considering why adolescents mentioned that a mental health professional should be the one who listens to them and accepts them, there is an assumption that maybe young people do not get that from their close environment (family or friends). On the other hand, perhaps the experiences that adolescents had were not satisfactory to them, since professionals often tend to look for the causes of problems according to certain protocols or symptom checklists, instead of listening only to the problems or concerns expressed by the adolescent.

However, when assessing mental health, the professionals are distinct in that they look from the perspective of the presence or absence of disorders. A systematic review by Radez et al. [25] found that in research on child and adolescent mental health, 30% of studies focused on general mental health, while the majority addressed specific mental health problems such as depression, anxiety, suicidal ideation, or ADHD. This leads to a situation where psychologists and psychiatrists mainly pay attention to particular symptoms and thus may not discuss the things that are relevant to adolescents in ‘plain language’, such as talking about loneliness, self-exclusion, and self-destruction.

### 4.1. Strengths and Limitations

Several strengths and limitations of this study should be mentioned. One of the advantages is that the problem was analyzed using a qualitative study design. This design enabled us to discover not only the relevance of certain mental health symptoms but also the subjective causes of certain phenomena. In this study, we specifically wanted to find out the perception of adolescents themselves about mental health—what is important to them, what are the presumed causes and effects. Additionally, our sample was sufficiently diverse and balanced in terms of age, gender, and residence to reveal a wider range of adolescents’ experiences and perceptions. This provides experiences of living in different contexts.

However, the major limitation of this study is the relatively small sample, so its findings should be generalized with caution. Thus, large-scale representative studies would enable checking the applicability of these findings in the general adolescent population. Moreover, only volunteers participated in our study, so the findings may be less representative of shy or less reflective adolescents.

The context of the COVID-19 pandemic when this study was conducted may also have affected the results, even though there were no interview questions related to the pandemic or isolation. Nonetheless, ample evidence was published during the pandemic showing that it had affected adolescent mental health. To see if the pandemic had an impact on mental health perception as a construct, post-pandemic research is needed. More so, given the qualitative nature of our study, checking its findings with a quantitative design may provide more robust conclusions.

### 4.2. Recommendations

A growing number of adolescents with psychological problems and mental health issues [48] shows a greater need for society and specialists to help this group of people. Especially given that mental health issues are becoming the “new normal” but still carry some stigma [17]. Therefore, to achieve the best goal and create the most targeted interventions, it is important to consider the adolescents themselves and their opinions. The results of this study could be used as a guideline to assess the potentially significant factors that influence adolescent mental health. Specifically, psychoeducation, family-school-community collaboration, and policy efforts are crucial to meeting the mental health service needs of adolescents with mental health problems.

## 5. Conclusions

Most adolescents define mental health as the ability to control their emotions and adjust their behavior according to societal and age norms, which is reflected in the quality of their relationships with other people. Assessment of adolescent mental health is also revealed through essential needs: supportive communication with significant people, a sense of a safe and private space, and the opportunity to engage in pleasant activities that help self-expression. Adolescents single out bullying and un-pleasant and annoying mutual communication from peers or adults as the most harmful factors for mental health. At the same time, time spent on social media and its harmful content can affect adolescents’ perceptions of the environment and themselves. Key risk factors that can help predict poor adolescent mental health include withdrawal, non-social behavior, and risky substance- or self-harm-related behaviors. To strengthen mental health, adolescents play a significant role in mental health care. Teenagers expect from professionals simple, un-hurried communication and a detailed conversation about important, significant aspects of life for a teenager.

## Figures and Tables

**Table 1:** The main characteristics of a study sample.

Characteristic	Value	*n*	%
Gender	Boy	9	47.4
	Girl	10	52.6
Age group	11–13 years	8	42.1
	14–15 years	6	31.6
	16–17 years	5	26.3
Grade	4–6	9	47.4
	8–9	6	31.6
	10–11	4	21.1

## Data Availability

The data presented in this study are available on request from the corresponding author. The data are not publicly available due to their containing information that could compromise the privacy of research participants.
